# Supplementary material for: Getting More Out of Biomedical Documents with GATE's Full Lifecycle Open Source Text Analytics
Source: PLoS Comput Biol. 2013 Feb 7;9(2):e1002854. doi: 10.1371/journal.pcbi.1002854 (PMC3567135; doi:10.1371/journal.pcbi.1002854)
Supplement: Dataset S1 — GATE software. Dataset S1 bundled with this paper contains a distribution of GATE (or see http://gate.ac.uk/download/). (TGZ) [file pcbi.1002854.s001.tgz › plos-gate/plugins/Tagger_MetaMap/doc/info/olteanu/utils/TextNormalizer.Java5Normalizer.html]

TextNormalizer.Java5Normalizer


---


|  |  |  |
| --- | --- | --- |
| **Overview** \| **Package** \| **Class** \| **Use** \| **Tree** \| **Deprecated** \| **Index** \| **Help** | |  |
| **PREV CLASS**   **NEXT CLASS** | **FRAMES**    **NO FRAMES**     **All Classes** |  |
| SUMMARY: NESTED \| FIELD \| CONSTR \| METHOD | DETAIL: FIELD \| CONSTR \| METHOD |  |


---


## info.olteanu.utils Class TextNormalizer.Java5Normalizer

```
java.lang.Object
  info.olteanu.utils.TextNormalizer.Java5Normalizer
```

**All Implemented Interfaces:**: StringFilter

**Enclosing class:**: TextNormalizer

---

``` public static class TextNormalizer.Java5Normalizer extends java.lang.Object implements StringFilter ```

---

**Field Summary**

**Fields inherited from interface info.olteanu.interfaces.StringFilter**

`INVALID, VOID`


**Constructor Summary**

`TextNormalizer.Java5Normalizer()`


| **Method Summary** | |
| --- | --- |
| `java.lang.String` | `filter(java.lang.String text)` |

**Methods inherited from class java.lang.Object**

`clone, equals, finalize, getClass, hashCode, notify, notifyAll, toString, wait, wait, wait`

**Constructor Detail**

### TextNormalizer.Java5Normalizer

```
public TextNormalizer.Java5Normalizer()
                               throws java.lang.ClassNotFoundException
```

**Throws:**: `java.lang.ClassNotFoundException`


**Method Detail**

### filter

```
public java.lang.String filter(java.lang.String text)
```

:   **Specified by:**: `filter` in interface `StringFilter`


---


|  |  |  |
| --- | --- | --- |
| **Overview** \| **Package** \| **Class** \| **Use** \| **Tree** \| **Deprecated** \| **Index** \| **Help** | |  |
| **PREV CLASS**   **NEXT CLASS** | **FRAMES**    **NO FRAMES**     **All Classes** |  |
| SUMMARY: NESTED \| FIELD \| CONSTR \| METHOD | DETAIL: FIELD \| CONSTR \| METHOD |  |


---
